# More Than Cell Markers: Understanding Heterogeneous Glial Responses to Implantable Neural Devices

**DOI:** 10.3389/fncel.2021.658992

**Published:** 2021-04-12

**Authors:** Ouzéna Bouadi, Tuan Leng Tay

**Affiliations:** ^1^Faculty of Biology, University of Freiburg, Freiburg, Germany; ^2^Faculty of Life Sciences, University of Strasbourg, Strasbourg, France; ^3^BrainLinks-BrainTools Centre, University of Freiburg, Freiburg, Germany; ^4^Freiburg Institute of Advanced Studies, University of Freiburg, Freiburg, Germany

**Keywords:** neural probe, microglia, astrocytes, oligodendrocytes, heterogeneity, neuroinflammation, acute brain injury, foreign body response

## Introduction

Recent publicity surrounding a coin-size computer chip in a pig's brain has placed the spotlight on the field of neurointerfaces (Lewis, 2020). Implantable microelectrode arrays (MEAs), or neural probes, enable the study of brain activity and present promising treatment and therapeutic options for neurological conditions (Boehler et al., 2020). These range from motor and sensory impairments such as spinal cord injuries and hearing loss, to neuropsychiatric disorders including dementia, clinical depression and insomnia. Application-specific MEAs that, for example, record field potentials and neuronal activity have been validated in non-human primates and could help understand mechanisms underlying motor functions and epilepsy (Barz et al., 2017; Gerbella et al., 2021). Key design considerations for biocompatibility, efficacy and longevity of microelectrodes to maintain long-term neuronal recording and stimulation are highly dependent on brain tissue response (Polikov et al., 2005). The functional capacities of a biosensor depend on the number of surrounding neurons in a given radius (50–350 μm) (He et al., 2020). Probe insertions generate inflammatory responses to acute tissue injuries and the introduction of foreign bodies, known as “foreign body response” (FBR). Chronic neuroprosthetic implants in rats at 16 weeks in contrast to 8 weeks have been shown to increase neuronal and dendritic loss, correlate with tau hyperphosphorylation seen in Alzheimer's disease and other tauopathies, and impede regeneration and recording of activity surrounding the device (McConnell et al., 2009). Assessments of acute proinflammatory events and chronic progression have largely centered on histological analyses of non-neuronal central nervous system (CNS) cells such as microglia, astrocytes and oligodendroglia, including their contribution to neuroinflammation and glial scars (Kozai et al., 2015; Prodanov and Delbeke, 2016). However, immunohistochemistry provides qualitative answers and rarely discriminates between heterogeneous cellular states (Wellman et al., 2019). Here we highlight developments that expand our knowledge of context-dependent heterogeneity of glia and blood-brain barrier cells, proposing new approaches to examine the diverse contributions of non-neuronal CNS cells after probe implantation. Having a holistic understanding of multiple glial responses will advance neuroengineering that temper neuroinflammation and tissue scarring, thereby improving functional neuroprosthetic integration.

## Microglia at the Brain-Machine Interface

Microglia are myeloid cells of extra-embryonic origin that form the brain-resident macrophages (Ginhoux et al., 2010). Tissue damage triggers microglia-driven repair mechanisms including phagocytosis of cellular debris, chemotaxis, and initiation of cell death pathways through cytokine release (Prinz et al., 2019). As first responders of the CNS that potentially contribute to sustained neuroinflammation, microglial reactivity is widely assessed after microelectrode implantation to examine changes elicited by insertion injury and FBR (Kozai et al., 2012). Intracortical implantation of non-functional microelectrodes in rats has led to the elevation of oxidative stress markers (Ereifej et al., 2018). Microglia have been shown to increase acidosis and inflammation by the release of reactive oxygen species (ROS) in a controlled cortical impact (CCI) mouse model for neurotrauma (Ritzel et al., 2021). ROS can be detrimental to long-term functionality of an implanted sensor (Takmakov et al., 2015). Prolonged microglial reactivity or adherence to the electrode surface could threaten device efficacy and longevity in the recipient brain and diminish recording quality (Huang et al., 2020). Moreover, microglia secretion of proinflammatory cytokines such as tumor necrosis factor (TNF) and interleukin 1 (IL-1) may induce neurotoxic reactive astrocytes (Liddelow et al., 2017) to envelope the implant. Together with cell recruitment, glial scar formation and electrode encapsulation, proinflammatory microglia have earned the reputation of being noxious (Kozai et al., 2016). Yet depletion of microglia was shown to be unfavorable for scar formation, wound healing and survival of neurons and oligodendrocytes (Bellver-Landete et al., 2019), supporting the notion that they promote the stable integration of implanted MEAs. Microglial heterogeneity in the healthy, developing and diseased brain is very well-described (Stratoulias et al., 2019; Masuda et al., 2020), even if mammalian microglia mostly originate from a single erythromyeloid progenitor source in the embryonic yolk sac (Alliot et al., 1999; Ginhoux et al., 2013). However, prevailing studies of MEAs do not reveal the spectrum of neuroprotective or neurotoxic microglial subtypes.

**Figure 1:**
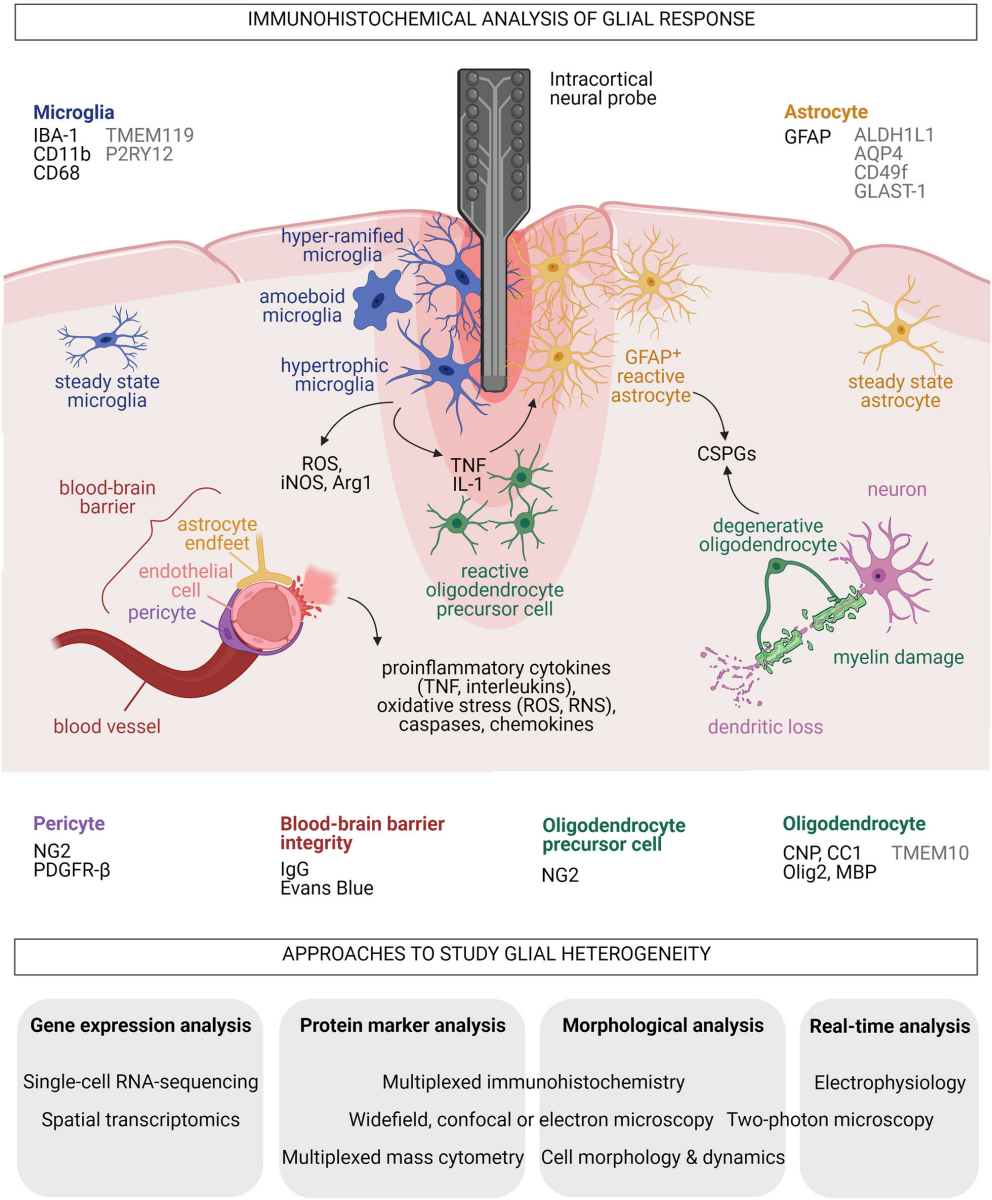
Summary of acute glial responses to implantation of neural microprobes in the mammalian brain and proposed approaches to study glial heterogeneity. Common markers (black) and additional markers (gray) for histological analyses of each glial type and blood-brain barrier integrity around the implants are listed. Black arrows indicate secreted molecules. Created with BioRender.com.

Common markers for microglia and microglial reactivity, such as ionized calcium-binding adaptor molecule 1 (IBA-1), integrin alpha M (ITGAM, or CD11b) and CD68 (also ED-1), are frequently used in immunohistochemical analysis of the brain-electrode interface as readout for tissue damage caused by implantation trauma and FBR (McConnell et al., 2009; Luan et al., 2017; Huang et al., 2020) (Figure 1). Transmembrane protein 119 (TMEM119) (Bennett et al., 2016) and purinergic receptor P2Y12 (P2RY12) (Butovsky et al., 2014) are excellent markers for homeostatic microglia, but are thus far rarely used for neurointerfaces. Neuroengineers considered the normalized intensity of microglial cell markers to proportionately represent the degree of inflammation (Lo et al., 2018). For instance, signal intensities of microglial cell markers surrounding insertion sites of explanted probes were examined at acute (1–3 days) or sub-chronic (up to 28 days) phases to evaluate the brain-machine interface (Lind et al., 2013; Wellman et al., 2019). Studies on neurotrauma and FBR showed that microglia could upregulate proinflammatory inducible nitric oxide synthase (iNOS) (Madathil et al., 2018) or anti-inflammatory arginase 1 (Arg1) (Sawyer et al., 2014). Descriptions of microglial cell morphologies in the assessment of FBR after implantation of MEAs include “ramifying” and “amoeboid,” which are, respectively, associated with steady and reactive states (Huang et al., 2020). Additional classifications such as “primed,” “hypertrophic” and “hypo- or hyper-ramified” are also relevant for the phenotypic characterization of neuroprotective or neurotoxic microglia in pathological conditions (Verdonk et al., 2016). Current immunohistochemical analyses however mostly disregards microglial diversity at the implantation site.

## Astrocytes and Scarring at Neurointerfaces

Astrocytes are star-shaped, heterogeneous glial cells that provide significant neurotrophic support through their interaction with every component of the CNS parenchyma (Verkhratsky and Nedergaard, 2018). They support synapse formation, maturation and pruning, and modulate pre- and post-synaptic transmission in homeostatic CNS (Sofroniew and Vinters, 2010). Tissue damage unleashes reactive astrocytes that adopt neuroprotective or neurodegenerative properties (Liddelow and Barres, 2017). Glial fibrillary acidic protein (GFAP) is the most frequently used immunohistochemical marker for reactive astrocytes in analyses of brain-electrode interface and is positively correlated to astrogliosis and glial scar formation (Polikov et al., 2005; Seymour and Kipke, 2007; Kozai et al., 2015; Prodanov and Delbeke, 2016) (Figure 1). GFAP^+^ astrocytes contribute to scarring through secretion of extracellular matrix chondroitin sulfate proteoglycans (CSPGs) such as neurocan, phosphacan and brevican (Fawcett and Asher, 1999; Matsui et al., 2002). CSPGs are inhibitors of axonal growth and remyelination that are frequently found in multiple sclerosis lesions where they reduce adherence of oligodendrocyte precursor cells (OPCs) for myelin repair (Galtrey and Fawcett, 2007; Lau et al., 2012). Inserting pieces of nitrocellulose filter into adult rat brain cortices induced infiltration of GFAP^+^ astrocytes into the implants and continued release of CSPGs even at 1 month after tissue injury (McKeon et al., 1991, 1999). Reactive astrocytes formed the principal cell type that increasingly compacted around and encapsulated a silicon microprobe implanted for up to 12 weeks in rats (Turner et al., 1999). This was similarly observed in a marmoset brain carrying an array with 32 Teflon-coated 50-μm-large microelectrodes for 7 months (Budoff et al., 2019). High levels of CSPGs were concomitantly observed with neuronal loss after an uncoated silicon neural probe was implanted in rat brains (Zhong and Bellamkonda, 2007). Recording performance of multichannel, 16-shank, silicon “Utah” MEAs embedded year-long in feline sensorimotor cortex reportedly dropped when neuronal action potentials were recorded (McCreery et al., 2016). This implicates astrocytic glial scar and neuronal death in the loss of biosensor performance. However, reactive astrocytes unlikely lead to only destructive outcomes. Conditional ablation of astrocytes after CCI, stab or crush injuries augmented lesion formation, demyelination and death of neurons and oligodendrocytes (Faulkner et al., 2004; Myer et al., 2006). The multifaceted roles of astrocytes suggest they are also vital promoters of repair.

Elucidating long-term changes in astrocytic FBR and scarring at neuroprosthetic implantation sites requires an understanding of astrocyte heterogeneity. Astrocytic diversity is well-described in healthy, developing and diseased CNS (Khakh and Sofroniew, 2015; Chai et al., 2017; Lanjakornsiripan et al., 2018; Clavreul et al., 2019; Pestana et al., 2020) and cannot be represented by the GFAP marker. Markers for homeostatic astrocytes include aldehyde dehydrogenase 1 family member L1 (ALDH1L1) (Cahoy et al., 2008), glutamate aspartate transporter 1 (GLAST-1, also known as excitatory amino acid transporter 1, EAAT-1) (Hurwitz et al., 1993) and aquaporin-4 (AQP4) found in astrocytic end feet (Yoneda et al., 2001) (Figure 1). A newly described population of human induced pluripotent stem cells-derived, proinflammatory cytokine-stimulated reactive astrocytes specifically upregulate CD49f (Barbar et al., 2020). From gray matter protoplasmic astrocytes to white matter fibrous astrocytes, diverse astrocytic morphologies in the healthy and diseased brain are well-documented (Zhang and Barres, 2010; Molofsky et al., 2012; Bardehle et al., 2013; Bayraktar et al., 2014). Immunohistochemical analyses to date however exclude the heterogeneity of astrocytes surrounding an implant.

## Impact of Neuroprosthetics on Oligodendrocytes and Their Progenitors

Differentiation of OPCs, also known as NG2-glia, gives rise to oligodendrocytes that produce and maintain myelin sheaths, which provide neurotrophic support and optimize brain electrical signaling (Bradl and Lassmann, 2010; Nave and Werner, 2014). OPCs and newly derived oligodendrocytes are essential for remyelination and CNS repair following demyelinating diseases or brain injury (Young et al., 2013; Bechler et al., 2015). Immunohistochemical analyses of oligodendrocytes at neurointerfaces have involved markers including 2′,3′-Cyclic-nucleotide3′-phosphodiesterase (CNP) (Chen et al., 2021) and CC1 (a monoclonal antibody against adenomatous polyposis coli) for mature oligodendrocytes, oligodendrocyte transcription factor 2 (Olig2) for immature oligodendrocytes, and myelin basic protein (MBP) for myelinating oligodendrocytes (Wellman et al., 2018, 2019) (Figure 1). TMEM10, a type 1 transmembrane glycoprotein, was recently verified to be specific for mammalian CNS myelin (Golan et al., 2008; de Faria et al., 2019).

With limited antioxidant capacity and high iron content, oligodendrocytes are sensitive to elevated ROS and reactive nitrogen species (RNS) levels arising from glial response to acute implantation injury and FBR (Smith et al., 1999). Similar to GFAP^+^ astrocytes, OPCs and oligodendrocytes release axonal growth-inhibiting CSPGs including NG2 and myelin-associated glycoprotein (Fawcett and Asher, 1999). Studies on passive multi-channel, four-shank “Michigan” MEAs in murine visual cortex revealed acute oligodendrocyte injury and degeneration, myelin degradation, and reactive swarming of OPCs toward the implant within 12 h (Wellman and Kozai, 2018; Chen et al., 2021). Severe reduction of electrophysiological recording quality from neurons at various tissue depths and observations of decreased neuronal firing in a mouse model of demyelination highlighted the importance of myelin integrity for microelectrode function (Wellman et al., 2020). A clearer picture of the renewal, maturation and function of various oligodendroglia at implantation sites will allow to determine the degree of cohesiveness at the brain-machine interface.

## Impact of Neuroprosthetics on Blood-Brain Barrier Integrity

A breach of the blood-brain barrier (BBB) is inevitable during implantation for microelectrodes to reach the neurons. The BBB is composed of endothelial cells, pericytes, and astrocytes, forming the neurovascular unit together with surrounding microglia and neurons (Sweeney et al., 2016; Bennett et al., 2019). Cerebrovascular endothelial cells are seamlessly joined by active protein complexes known as tight and adherens junctions (Tietz and Engelhardt, 2015). Pericytes also regulate BBB permeability and are involved in neuroinflammatory response, clearance of toxic metabolites and promotion of angiogenesis (Hill et al., 2014). Pericytes are typically identified by the colocalization of NG2 and platelet-derived growth factor receptor beta (PDGFR-β) markers (to differentiate them from NG2^+^ OPCs) (Wellman et al., 2019) (Figure 1). Vasculature integrity is commonly assessed by histological detection of immunoglobulin G leakage or Evans blue staining for plasma membrane damage (Nolta et al., 2015; Falcone et al., 2019) (Figure 1). Decreased expression of junctional proteins in the compromised BBB promotes neuroinflammation through higher expression of proinflammatory cytokines and chemokines and increased infiltration of peripheral immune cells (Marchetti and Engelhardt, 2020). BBB leakiness, astrogliosis and neuronal death in brain tissue surrounding the implanted device were shown to reduce the number of measurable electrophysiological responses of single neurons and degrade the overall recording performance of the biosensor (Nolta et al., 2015). High-speed pneumatic intracortical insertion of Utah MEA in rat cortex has led to down-regulation of endothelial tight and adherens junction protein markers, and correlated with increased oxidative stress and elevated inflammation levels indicated by upregulation of caspases, chemokines, interleukins and TNF (Bennett et al., 2018, 2019) (Figure 1). Notably, BBB release of ROS, RNS, and proinflammatory cytokines and chemokines likely promote microglial and astrocyte reactivity, and loss of neurons and oligodendrocytes.

## Current Approaches to Study Glial Heterogeneity

Single-cell transcriptomic technologies that simultaneously quantify hundreds or thousands of expressed genes of individual cells in a given population have unmasked heterogeneous cellular identities and developmental trajectories, and revealed biomarker information (Aldridge and Teichmann, 2020). These powerful methods provide unique insights into health and disease in contrast to bulk transcriptomic and classical histological analyses. Single-cell RNA-sequencing and spatial transcriptomic approaches have shown that glia isolated from healthy and disease-associated brain regions respond across broad cellular states for microglia (Tay et al., 2018; Hammond et al., 2019; Jordão et al., 2019; Li et al., 2019; Masuda et al., 2019), astrocytes (Cahoy et al., 2008; Zamanian et al., 2012; Boisvert et al., 2018; Bradley et al., 2019; Batiuk et al., 2020; Bayraktar et al., 2020; Das et al., 2020), and oligodendrocytes (Jäkel et al., 2019; Spitzer et al., 2019; Floriddia et al., 2020). Advances in single-cell proteomics have also enabled the high-throughput investigation of key biological questions involving protein binding, modifications, and degradation, that cannot be assessed at the transcriptomic level (Slavov, 2020). Multiplexed mass cytometry and multiplexed immunohistochemistry have unveiled regional and pathology-dependent heterogeneity of human peripheral myeloid cells, microglia and astrocytes (Böttcher et al., 2019; Park et al., 2019). Furthermore, multiplexed immunohistochemistry, electron microscopy and *in vivo* two-photon imaging techniques are increasingly applied to study acute and chronic oligodendrocyte and OPC reactivity after microprobe implantation (Bogoslovsky et al., 2018; Michelson et al., 2018; Wellman and Kozai, 2018; Chen et al., 2021) (Figure 1). Clearly, advancing MEA technology requires a comprehensive examination of glial responses at neurointerfaces by integrating quantitative single-cell multi-omic analyses with assessments of cell morphology and dynamics, and electrophysiological recordings, as has been recently demonstrated in neurons (Cadwell et al., 2016).

## Discussion

Implantation of single-shank or multi-shank MEAs will inevitably trigger changes in glia and the BBB due to acute tissue trauma and FBR. Microglia surrounding the lesion will immediately undergo significant state changes to limit physical damage through microgliosis, phagocytosis of dying cells and debris, and release of proinflammatory cytokines and stress-induced molecules. Microglial reactivity likely elevates the population of reactive astrocytes, which could lead to extensive unwanted glial scarring. In concert with astrocytes, degenerative oligodendrocytes also secrete growth-inhibiting extracellular matrix components, and result in electrode encapsulation and dysfunction. Loss of BBB homeostasis also exacerbates proinflammatory responses of microglia and astrocytes to favor neuronal and myelin loss. CNS repair however necessitates acute inflammatory events contributed by neuroprotective subpopulations of non-neuronal brain cells. To harness the endogenous, neuro-regenerative properties of glia and promote electrode biocompatibility and longevity (Gulino et al., 2019), we propose to investigate context-dependent glial responses at brain-machine interfaces using combinatorial approaches in addition to immunohistochemical assays of protein markers. Probe fabrication breakthroughs in material, size and geometry have limited implantation trauma and reduced probe encapsulation (Patel et al., 2016; Luan et al., 2017; Rivnay et al., 2017). Devices coated with dexamethasone to alleviate neuroinflammation (Kozai et al., 2016; Boehler et al., 2017), or laminin to restrict glial reactivity at implantation sites (He et al., 2006), have shown great promise. A deeper molecular understanding of diverse glial responses at neurointerfaces will identify further candidates for promoting neuroprosthetics development.

## Author Contributions

OB wrote the first draft of the manuscript and designed the figure. TLT supervised the project and extensively revised the manuscript. All authors contributed to the article and approved the submitted version.

## Conflict of Interest

The authors declare that the research was conducted in the absence of any commercial or financial relationships that could be construed as a potential conflict of interest.
